# Nintedanib antiangiogenic inhibitor effectiveness in delaying adenocarcinoma progression in Transgenic Adenocarcinoma of the Mouse Prostate (TRAMP)

**DOI:** 10.1186/s12929-017-0334-z

**Published:** 2017-05-12

**Authors:** Raquel Frenedoso da Silva, Ellen Nogueira-Pangrazi, Larissa Akemi Kido, Fabio Montico, Sarah Arana, Dileep Kumar, Komal Raina, Rajesh Agarwal, Valéria Helena Alves Cagnon

**Affiliations:** 1 Department of Structural and Functional Biology, Institute of Biology, University of Campinas (UNICAMP), P.O. Box 6109, 13083-865 Campinas, São Paulo Brazil; 2 Department of Biochemistry and Tissue Biology, Institute of Biology, University of Campinas (UNICAMP), Campinas, São Paulo Brazil; 3 Department of Pharmaceutical Sciences, Skaggs School of Pharmacy, University of Colorado Anschutz Medical Campus, Aurora, CO USA

**Keywords:** Nintedanib, Angiogenesis inhibitor, Hormone receptors, VEGF, Prostate cancer, TRAMP

## Abstract

**Background:**

In recent times, anti-cancer treatments have focused on Fibroblast Growth Factor (FGF) and Vascular-Endothelial Growth Factor (VEGF) pathway inhibitors so as to target tumor angiogenesis and cellular proliferation. One such drug is Nintedanib; the present study evaluated the effectiveness of Nintedanib treatment against in vitro proliferation of human prostate cancer (PCa) cell lines, and growth and progression of different grades of PCa lesions in pre-clinical PCa *transgenic adenocarcinoma for the mouse prostate* (TRAMP) model.

**Methods:**

Both androgen-independent (LNCaP) and androgen-dependent (PC3) PCa cell lines were treated with a range of Nintedanib doses for 72 h, and effect on cell growth and expression of angiogenesis associated VEGF receptors was analyzed. In pre-clinical efficacy evaluation, male TRAMP mice starting at 8 and 12 weeks of age were orally-fed with vehicle control (10% Tween 20) or Nintedanib (10 mg/Kg/day in vehicle control) for 4 weeks, and sacrificed immediately after 4 weeks of drug treatment or sacrificed 6–10 weeks after stopping drug treatments. At the end of treatment schedule, mice were sacrificed and ventral lobe of prostate was excised along with essential metabolic organ liver, and subjected to histopathological and extensive molecular evaluations.

**Results:**

The total cell number decreased by 56–80% in LNCaP and 45–93% in PC3 cells after 72 h of Nintedanib treatment at 2.5–25 μM concentrations. In pre-clinical TRAMP studies, Nintedanib led to a delay in tumor progression in all treatment groups; the effect was more pronounced when treatment was given at the beginning of the glandular lesion development and continued till study end. A decreased microvessel density and VEGF immunolocalization was observed, besides decreased expression of Androgen Receptor (AR), VEGFR-1 and FGFR-3 in some of the treated groups. No changes were observed in the histological liver analysis.

**Conclusions:**

Nintedanib treatment was able to significantly decrease the growth of PCa cell lines and also delay growth and progression of PCa lesions to higher grades of malignancy (without inducing any hepatotoxic effects) in TRAMP mice. Furthermore, it was observed that Nintedanib intervention is more effective when administered during the early stages of neoplastic development, although the drug is capable of reducing cell proliferation even after treatment interruption.

## Background

Prostate cancer (PCa) is the most common type of cancer in men and the second leading cause of cancer-associated deaths, particularly in men over 50 years of age [1]. According to Siegel [2], more than 1.6 million new cases of PCa were diagnosed in 2015 in the United States alone. Genetic aberrations, capable of disrupting homeostasis between the epithelial and the stromal prostate compartments, are considered as one of the leading causes of this disease; prostatic stroma is responsible for the quick response in case of tissue injury/damage to the prostatic epithelium [3, 4].

Different animal models have been used in anti-PCa efficacy studies, including the *transgenic adenocarcinoma for the mouse prostate* (TRAMP) model, which mimics the spontaneous growth and progression of PCa as it occurs in humans. According to Wikstrom et al. [5], lesions found in the prostate of the TRAMP mice can be classified into different grades histopathologically, low and high grade prostatic intraepithelial neoplasia (PIN) lesions which advance to different stages of adenocarcinoma, such as well-differentiated, and poorly differentiated adenocarcinoma; in addition there are extensive changes in the expression of molecular markers [6]. The PIN stage is characterized by a stratification pattern and epithelial cell projection into the acinar lumen, showing atypical cells and cell polarity loss, nuclear increase, and chromatin condensation. Well-differentiated adenocarcinoma is characterized by the invasion of epithelial cells in the prostatic stroma and basement membrane disruption. This latter grade lesion can develop into poorly differentiated adenocarcinoma, where the tumor is made up of a cluster of indistinct cells [7].

Though the transgene is significantly expressed in dorsolateral prostate of TRAMP mice, it is also expressed at higher levels in the prostate ventral lobe [8]; a recent study has shown changes in the expression of 36 proteins during carcinogenesis in the ventral lobe of prostate [9]. Furthermore, a 2016 study showed a significant delay in tumor progression in the prostate ventral lobe of TRAMP mice after anti-inflammatory therapy [10].

Angiogenesis is known for its importance in the development and maintenance of the tumor and is responsible for the recruitment of new blood vessels from pre-existing vessels, occurring in response to the demand of nutrients and oxygen by tumor cells [11]. Currently, inhibition of tumor angiogenesis has been shown to be a promising therapeutic strategy in cancer treatment, and Vascular-Endothelial Growth Factor (VEGF) inhibitory drugs have been used successfully in clinical practice [12]. However, cancer cells may show a signaling exchange mechanism with the Fibroblast Growth Factor (FGF) pathway, leading to tumor growth even under VEGF inhibition. FGF signaling and receptors are responsible for regulating mechanisms such as differentiation, survival, motility and invasiveness, as well as being intimately involved in angiogenesis [13].

Currently Nintedanib (BIBF 1120), a selective FGF and VEGF receptor inhibitor, is being evaluated in clinical trials for its safety and efficacy against PCa treatment [13]. Studies have shown that Nintedanib is related to a significantly improved survival rate in patients [14]. Other studies have shown that Nintedanib administered at doses of 50–100 mg/Kg/day for 2 weeks could inhibit hepatocellular tumor growth in nude mice [15]. Furthermore, Nintedanib has been also shown to decrease tumor volume in mice injected with head, neck, and renal carcinoma cells [16]. Thus, the objective in the present study was to evaluate the efficacy of Nintedanib treatment against in vitro proliferation of human PCa cell lines, and the growth and progression of different grades of PCa lesions in TRAMP model. Besides investigating the effect on aberrant signaling pathways associated with PCa, the therapy effectiveness would also be analyzed on the structural and hormonal responses as well as the neovascularization of the prostate ventral lobe of TRAMP mice at different stages of the disease.

## Methods

### Reagents and cell culture

Human prostate carcinoma LNCaP and PC3 cells were obtained from American Type Culture Collection (Manassas, VA). Cells were cultured in RPMI 1640 with 10% fetal bovine serum (Hyclone, Logan, UT) under standard culture conditions (37 °C, 95% humidified air and 5% CO_2_). Cells were plated at a density of 5 × 10^3^ cells/cm^2^ plates under standard culture conditions. After 24 h, cells were treated with either DMSO alone (control), or different doses of Nintedanib dissolved in DMSO (concentration did not exceed 0.1% in any treatment (v/v)). At the end of treatment time (72 h), cells were collected after brief trypsinization, washed with PBS, and then stained with Trypan blue. The total cell number (colorless and blue stained) and dead cells (blue stained) were counted under light microscope using hemocytometer.

### Western blotting for cell lysates

Approximately 80 μg of protein lysate from whole-cell extracts per sample was denatured in 5 × sample buffer and subjected to sodium dodecyl sulfate–polyacrylamide gel electrophoresis (SDS-PAGE) on 8% Tris–glycine gel. The separated proteins were transferred on to nitrocellulose membrane followed by blocking with 5% non-fat milk powder (w/v) in Tris-buffered saline (10 mM Tris–HCl, pH 7.5, 100 mM NaCl, 0.1% Tween 20) for 1 h at room temperature. Membranes were probed for VEGFR-1 Vascular-Endothelial Growth Factor Receptor 1 - VEGFR-1 (sc-316) (Santa Cruz Biotechnology, USA) and VEGFR-2 (sc-6251) (Santa Cruz Biotechnology, USA) followed by the appropriate peroxidase-conjugated secondary antibody and visualized by ECL detection system. Membranes were also stripped and re-probed again with the loading control β-actin (A2228 Sigma-Aldrich). In each case, blots were subjected to multiple exposures on the film to make sure that the band density is in the linear range. The autoradiograms/bands were scanned with Adobe Photoshop 6.0 (Adobe Systems, San Jose, CA).

### Animals and experimental procedure

**Fig. 1:**
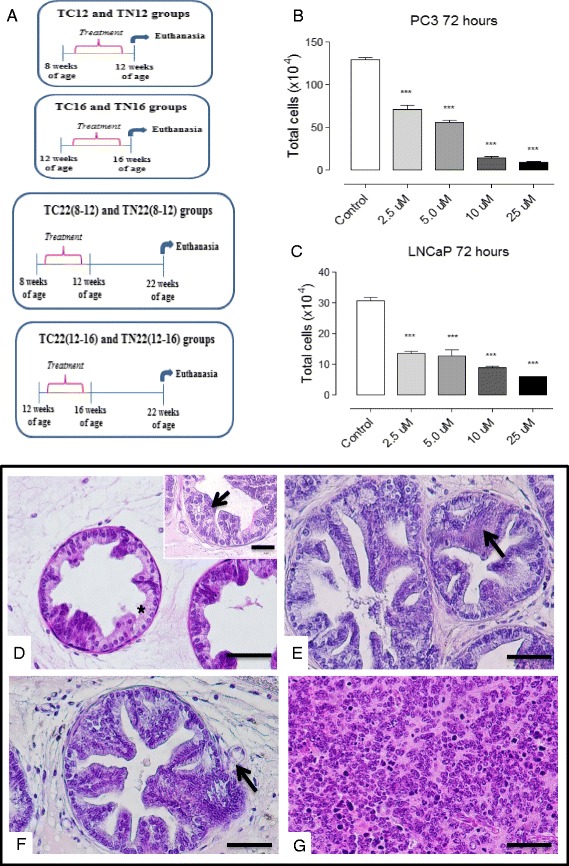
Experimental groups, cell viability and glandular lesion features of the prostate. **a** Scheme of the experimental design of the different groups **b** and **c** PC3 and LNCaP viability after Nintedanib treatment. **d** acini with healthy glandular areas/without lesion (asterisk) and low grade PIN occurrence (arrow). **e** high grade PIN (arrow), acini with lumen occupied by epithelial projections (cribriform type - Cr). **f** Epithelial cell invasion through the stroma with membrane basal discontinuity (arrow), characterizing well-differentiated adenocarcinoma. **g** Glandular disorganization with acini structure loss, characterizing undifferentiated adenocarcinoma. Hematoxylin and Eosin, 400x, bar = 100 μm

A total of 72 transgenic male mice - TRAMP (C57BL/6-Tg (TRAMP) 824Ng/JX FVB/JUnib) were provided by the Multidisciplinary Center for Biological Investigation in Laboratory Animal Science at the State University of Campinas (UNICAMP). The animals received water and solid food *ad libitum* (Nuvilab, Colombo, PR, Brazil). Procedures were approved by the Committee of Ethics in Animal Research (protocol n° 3285-1) and carried out in agreement with the Ethical Principles for Animal Research established by the Brazilian College for Animal Experimentation (COBEA). The mice were weighed and divided into 9 experimental groups (*n* = 10 per group), as follows (Fig. 1a), receiving either the vehicle (10% Tween 20, 10 mL/kg/day) or Nintedanib (10 mg/Kg/day in control vehicle) orally [16]: **T8 group:** untreated 8-week-old TRAMP mice; **TC12 and TN12 groups**: TRAMP mice treated with vehicle or Nintedanib, respectively, from 8 to 12 weeks of age and sacrificed thereafter; **TC16 and TN16 groups**: TRAMP mice treated with vehicle or Nintedanib, respectively, from 12 to 16 weeks of age and sacrificed thereafter; **TC22 (8–12) and TN22 (8–12) groups**: TRAMP mice treated with vehicle or Nintedanib, respectively, from 8 to 12 weeks of age and sacrificed 10 weeks later (22 weeks of mice age); **TC22 (12–16) and TN22 (12–16) groups**: TRAMP mice treated with vehicle or Nintedanib, respectively, from 12 to 16 weeks of age and sacrificed 6 weeks later (22 weeks of mice age). At the end of treatment, the mice were weighed on a *Denver P-214* scale (*Denver Instrument Company, Arvada, CO, EUA*) anesthetized with 2% xylazine hydrochloride (5 mg/Kg; Konig, São Paulo, Brazil) and 10% ketamine hydrochloride (60 mg/Kg; Fort Dodge, IA) and euthanized. Samples from the prostate ventral lobe were collected and submitted to histopathological, immunohistochemical and western blotting analyses. The body weight gain during the treatment (*n* = 3) were recorder for each experimental group.

### Histopathological analysis

Five prostate ventral lobe samples per experimental group were fixed in Bouin solution, washed in 70% ethanol, dehydrated in increasing concentration of ethanol, diaphanized in xylene and embedded in Paraplast (Paraplast Plus, St. Louis). Five μm thick sections were obtained using a microtome (Zeiss Hyrax M60) and the sections were deparaffinized in xylene, hydrated in a decreasing ethanol series, rinsed under distilled water and staining with hematoxylin–eosin. Photomicrographs were captured by a digital camera coupled to a Nikon Eclipse E-400 light microscope (Nikon, Tokyo, Japan). Morphological evaluation was performed (blind assay) in 15 randomly captured areas per animal (under 400x magnification), resulting in 75 areas per group. Each area was divided into 4 quadrants and histopathologically classified as per modified recommendations of Berman-Booty [7]: Normal, (without lesion), Low/High Grade Prostatic Intraepithelial Neoplasia (PIN) and Well-differentiated adenocarcinoma. Liver samples from TC12, TN12, TC22 (8–12) and TN22 (8–12) groups were also collected and subjected to same histopathological protocol as above.

### Immunohistochemistry

Tissue samples of prostate ventral lobe as were used in the histopathological analyses were also used for CD31, Androgen Receptor (AR), Estrogen Receptor α (ERα), Proliferating cell nuclear antigen (PCNA) and Vascular Endothelial Growth Factor (VEGF) immunohistochemical analysis using standard protocol. Briefly, antigens were retrieved by boiling the sections in 10 mM citrate buffer (pH 6.0). After that, the sections were incubated in H_2_O_2_ to block endogenous peroxidase. Nonspecific binding was blocked by incubating the sections in a blocking solution (BSA in TBS-T buffer) for 1 h at room temperature. AR, ERα, PCNA, CD31 and VEGF antigens were detected using polyclonal rabbit anti*-*AR (sc-816) (Santa Cruz Biotechnology, USA), polyclonal rabbit anti-ERα (sc-542) (Santa Cruz Biotechnology, CA), anti-PCNA [PC10] (ab29) Abcam for PCNA, polyclonal rabbit anti-CD31 (sc-1506-R) (Santa Cruz Biotechnology, CA) and polyclonal mouse anti-VEGF (sc-53462) (Santa Cruz Biotechnology, CA, USA), respectively, diluted in 1% BSA and applied to the sections overnight at 4 °C. The sections were then washed with TBS-T and subsequently incubated in HRP-conjugated secondary antibody from the Envision Kit (DAKO) for 60 min for CD31, goat anti-rabbit IgG (W4018) (Promega Corporation, Madison, WI) for AR and ERα and goat anti-mouse IgG (W4021) for VEGF detection. After washing in TBS-T, peroxidase activity was detected using a diaminobenzidine (DAB) chromogenic (Sigma-Aldrich, St. Louis, MO) for 5 min, which indicated the immunoreactivity of antibodies. Sections were lightly counterstained with Harris Hematoxylin, dehydrated in an increasing ethanol series and xylene, mounted in Entellan (Merck, Darmstadt, Germany) and photographed using the Nikon Eclipse E-400 microscope. The prostatic sections were analyzed using a multipoint system with 160 intersections [17]. Thus, 10 fields per mice, totaling 50 fields per group, were randomly captured (under 400x magnification). Values were determined by immunoreactivity count coinciding with the grid intersection divided by the total number of points. The result was expressed as a relative frequency of positive staining for the molecule in all experimental groups. Samples that were not incubated with primary antibody were used as negative controls.

### Microvessel density

Microvessel density determination was performed by counting the number of CD31 positive blood vessels in the prostatic stroma in ten random and non-overlapping fields per animal, following the criteria proposed by Weidner [18]. Microvessel density was expressed as the mean value obtained from the ten fields in each animal and also as the maximum density in a particular field (modified from Hochberg, [19]).

### Western blotting for tissue lysates

Prostate ventral lobe samples from five animals per group were collected and frozen in liquid nitrogen. The samples were weighed and homogenized in a Polytron homogenizer (Kinematica Inc., Lucerne, Switzerland) in a 40 mL/mg protein extraction buffer. The tissue homogenates were centrifuged at 18,659 rcf for 20 min at 4 °C and a sample of each extract was used for protein quantification with Bradford reagent (Bio-Rad Laboratories, Hercules, CA). The supernatants were mixed (1:1) with 3X Laemmli buffer and transferred to a dry bath at 100 °C for 5 min. Aliquots containing 50 or 75 μg of protein were separated by electrophoresis in SDS-PAGE gels under reducing conditions. After electrophoresis, proteins were transferred to Hybond-ECL nitrocellulose membranes (Amersham, Pharmacia Biotech, Arlington Heights, IL) at 120 V for 90 min. The membranes were blocked with BSA in TBS-T for 60 min and incubated at 4 °C overnight with the primary antibodies for AR, ERα and VEGF (described under the immunohistochemistry section) and Fibroblast Growth Factor Receptor 3 – FGFR-3 (sc-123) (Santa Cruz Biotechnology, USA) and Vascular-Endothelial Growth Factor Receptor 1 – VEGFR-1 (sc-9029) (Santa Cruz Biotechnology, USA). Membranes were then incubated for 2 h with the same HRP-conjugated secondary antibodies used for immunostaining diluted in a 1:5,000–1:10,000 range. After washing in TBS-T, peroxidase activity was detected through the incubation of the membranes in the chemiluminescent solution (Pierce Biotechnology, Rockford, IL) for 5 min, followed by fluorescence capture using the Gene Gnome equipment and the Gene Sys image acquisition software (Syngene Bio Imaging, Cambridge, UK). Mouse monoclonal anti-β-actin (sc-81178) (Santa Cruz Biotechnology, CA) antibody was used as an endogen control for comparison among groups. The intensity of antigen bands in each experimental group was determined by densitometry using the Image J (Image Analysis and Processing in Java) software for image analyses and was expressed as the mean percentage ± standard deviation in relation to β-actin band intensity.

### Statistical analysis

Data are presented as average percentage or mean ± Standard Error of Mean (SEM). Parametric variables were compared by ANOVA followed by the test of Bonferroni or by two-tailed *t-test* (for morphology and Western Blotting analysis). Differences were considered significant when $p$<0.05. The statistical analyses were performed by the software GraphPad Prism (version 5.0).

## Results

### Inhibitory effects of Nintedanib on growth of androgen–dependent and –independent human PCa cells

In order to evaluate the anticancer efficacy of Nintedanib against both androgen-dependent and –independent human PCa cells, we studied the in vitro effect of Nintedanib treatment on the cellular growth/viability of both cell types (under 10% serum conditions) using Trypan blue dye exclusion method. Both androgen-dependent LNCaP and androgen–independent PC3 cells treated with Nintedanib at the concentrations of 0, 2.5, 5, 10 and 25 μM for 72 h showed a concentration dependent decrease in growth of these cells. The total cell number decreased by 56–80% (*P* < 0.001) in LNCaP and 45–93% (*P* < 0.001) in PC3 cells after 72 h of Nintedanib treatment at 2.5– 25 μM concentrations, respectively (Fig. 1b and c). These in vitro observations confirmed that Nintedanib had inhibitory effect against the growth of both androgen-dependent and –independent human PCa cells.

### TRAMP mice as an appropriate model for prostate ventral lobe adenocarcinoma evaluation

**Fig. 2:**
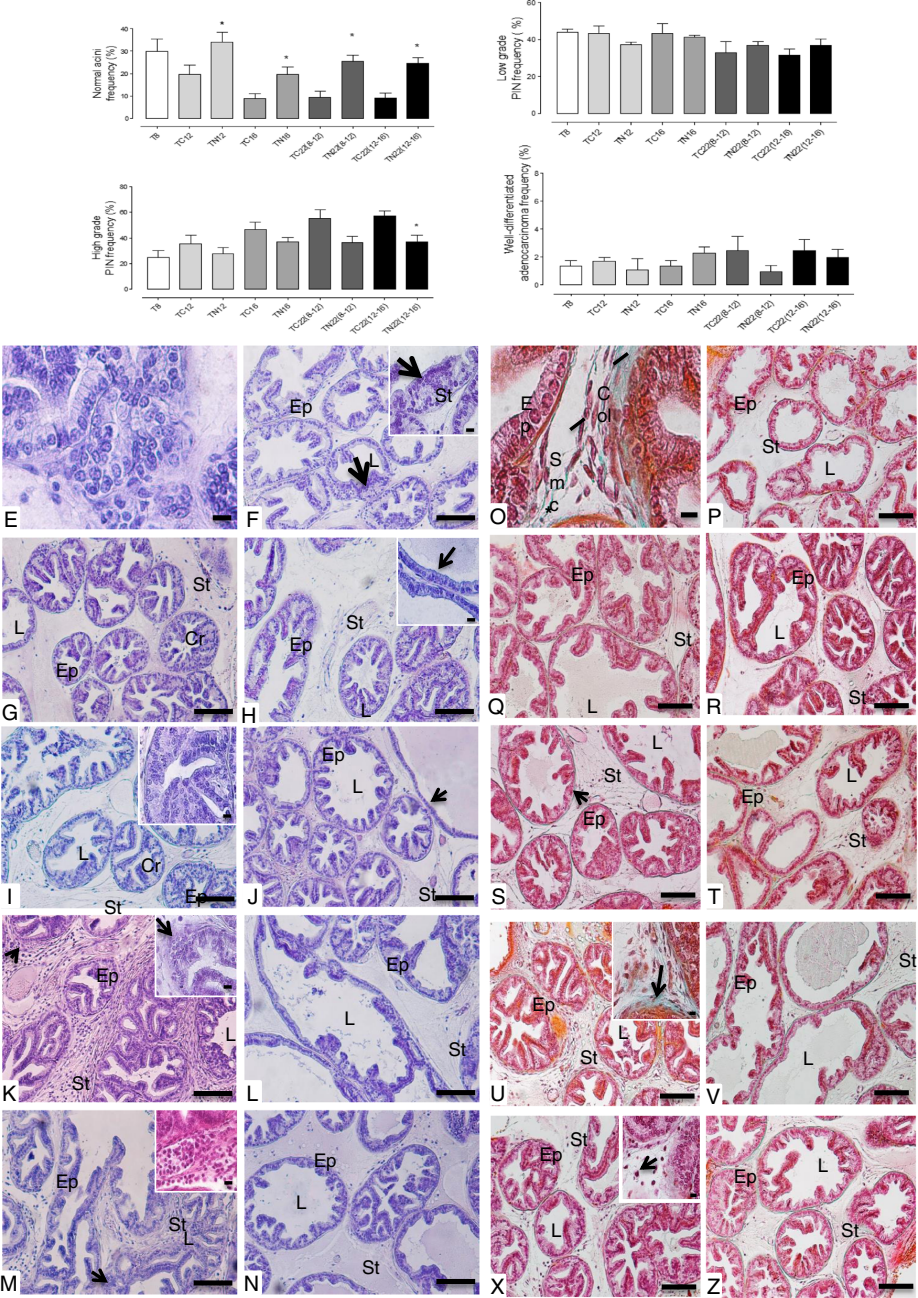
Frequency of the different lesion types and photomicrographs of the prostate ventral lobe. **a** Normal prostatic acini. **b** Low-grade PIN. **c** High-grade PIN and **d** Well-differentiated adenocarcinoma. Values expressed as average percentage. *p < 0.05 **e** atypical secretory epithelial cells in the prostate, highlighting cellular increase (1000x, bar = 20 μm). **f** T8 group – epithelial cell proliferation; low grade PIN foci (arrow); inset: 1000x, bar = 20 μm. **g** TC12 group – cellular proliferation with prostatic epithelium stratification, occupying acinus lumen, characterizing high grade PIN with cribriform feature acini (Cr); **h** TN12 group – epithelial atrophy and cellular proliferation scarcity (inset (1000x, bar = 20 μm)). **i** TC16 group – High grade PIN foci, cribriform feature acini (Cr and inset (1000x, bar = 20 μm)). **j** TN16 group – a slight decrease of cellular proliferation and epithelial atrophy (arrow). **k** TC22(8–12) group – cellular proliferation foci, characterizing well-differentiated adenocarcinoma (arrows); inset: 1000x, bar = 20 μm. **l** TN22(8–12) group and **n** TN22(12–16) group – Occasional cellular proliferation foci. **m** TC22(12–16) group – Cellular proliferation and infiltration in the stroma (inset), with well-differentiated adenocarcinoma foci (arrow). **o** accumulation of inflammatory cells and thickening of the collagen fiber layer (asterisk) (1000x, bar = 20 μm). **p** T8 group – normal distribution of fibrillar elements. **q** TC12 group – slight increase of fibrillar elements. **r** TN12 group – fibrillar stromal elements distributed regularly around the glandular acini. **s** TC16 group –a slight thickening of collagen fibers layer (arrow). **t** TN16 group – stroma without changes in the fibrillar and cellular elements. **u** TC22(8–12) and **x** TC22(12–16) groups – hyperplasia and hypertrophy of muscle cells, thickening of collagen fibers layer (U, arrow) and inflammatory cell accumulation (X, arrow) (inset (1000x, bar = 20 μm). **v** TN22(8–12) and **z** TN22(12–16) groups – occasional foci of inflammatory cells and layers of collagen fibers. Ep = epithelium; St = estroma; L = lumen; Cr = cribriform acinus, Col = collagen fibers; Smc = smooth muscle cells. Hematoxylin-Eosin and Masson Tricomic. (E to Z: 200x, bar = 200 μm. Insets: 1000x, bar = 20 μm)

Though most of the TRAMP studies have focused on efficacy evaluation in dorsolateral prostate, the transgene is also expressed in ventral prostate of TRAMP mice. In this study, we focused on neoplastic changes as observed in ventral prostate; this lobe also showed progressive development of PCa with increase in age in the TRAMP mice. The lesions were characterized as acinar epithelium proliferative lesions with different grades of severity. In 8- and 12-week-old mice (T8 and TC12 groups) there was a predisposition of low grade PIN (43.86% and 43.16%, respectively), characterized by cellular proliferation without occupying the lumen acinar known as the micro-papillary form (Figs. 1d, 2b, f and g), at the beginning of the prostatic lesion development. In these lesions, cellular atypia, such as the increase of nuclear size and cytoplasm reduction were verified in the epithelium (Fig. 2e). Also, the occurrence of inflammatory cells and hyperplasia of the smooth muscle cells were verified in the prostatic stroma. The smooth muscle cells were placed around the stroma, in addition to collagen fiber thickening, particularly around glandular regions showing epithelial cell proliferation (Figs. 2o, p and q). On the other hand, the predisposition of high grade PIN was verified in 16-week-old mice (TC16 group), representing 46.61%, showing higher frequency of epithelial stratification (Figs. 2c and e). The proliferative foci of prostatic epithelium in this group occupied the greater part of the acinar lumen, characterizing the acini with a cribriform feature (Fig. 1e). Finally, the 22-week-old mice TC22 (8–12) and TC22 (12–16) presented prostatic adenocarcinoma in advanced grades of development, characterized by the frequent occurrence of high grade PIN and representing 55.28% of the glandular total in the TC22 (8–12) group and 57.08% in the TC22 (12–16) group (Figs. 2c, k, and m). Also, there was well-differentiated adenocarcinoma characterized by epithelial cell proliferation invading the stroma, characterizing basal membrane discontinuity (Fig. 1e). In addition, undifferentiated adenocarcinoma was seen, characterized by acinar structure loss, in which the epithelial cells occupied the organ entirely (Fig. 1g). The prostatic stroma of these two groups also presented collagen fiber thickening, with the disorganization of stromal elements (Figs. 2u and x).

### Nintedanib delayed adenocarcinoma progression in the prostate ventral lobe, particularly when it was administered at the beginning of glandular lesion development

The mice from TN12 and TN16 groups showed late prostatic adenocarcinoma progression, characterized by a significant increase of healthy acinar frequency and decrease of high grade PIN foci in all Nintedanib treated groups vs. their control groups (Figs. 2a, c, h and j). On the other hand, the reduction of prostatic epithelial cell proliferation was higher in the TN12 group where Nintedanib treatment was given from 8 to 12 weeks of age and mice were sacrificed immediately after treatment end (Fig. 2h).

The mice from the TN12 group also showed a significant increase in a no-lesion acini frequency (34%), in addition to epithelial atrophy in comparison to the control group (TC12). Also, Nintedanib treatment reduced the well-differentiated adenocarcinoma incidence to 1.07%. There was also a reduction of low grade PIN (37.21%) and high grade PIN (27.71%) in relation to the TC12 control group (Figs. 2b and h). The prostatic stroma from the Nintedanib treated mice showed a slight decrease of fibromuscular layer thickening. However, hypertrophied smooth muscle cells, and regions with inflammatory cells were still observed (Fig. 2r). The TC12 group showed a predominance of low grade PIN (43.16%), while only 19.62% of the total glandular area showed acini without structural changes (Fig. 2b and g). High grade PIN foci (35.31%) and well–differentiated adenocarcinoma (1.68%) were verified in the TC12 group. The prostatic stroma in the mice from the TC12 group showed a slight fibrillar element increase, characterized by smooth muscle cell hypertrophy and collagen fiber layer thickening. In addition, inflammatory cells occurrence was identified, which was always seen around epithelial proliferation foci in the TC12 group prostate ventral lobe (Fig. 2q).

Furthermore, the prostate ventral lobe in the TN16 group showed a decrease in high grade PIN incidence (36.80%), in addition to a significant increase in glandular areas with healthy acini (19.72%) compared to control prostates in the TC16 group, which showed a predominance of high grade PIN (46.61%) (Fig. 2a, c, i and j). Also, slight collagen fiber layer thickening in the prostatic stroma was observed in the TN16 group in contrast to the pronounced thickening in the TC16 group (Fig. 2s and t).

### Nintedanib decreased adenocarcinoma frequency in the prostate ventral lobe despite stopping its treatment

The histopathological evaluation indicated that there was also delay in adenocarcinoma progression in the prostate ventral lobe of TRAMP mice whose Nintedanib treatments had ended 10 or 6 weeks before euthanasia (Fig. 2k, l, m and n). This effect was also associated with a significant increase of healthy prostatic area frequency and a decrease of high grade PIN frequency (Fig. 2a and c).

The prostate ventral lobe of the TN22 (8–12) group showed a reduction of high grade PIN frequency (36.59%) and an increase of healthy glandular areas and low grade PIN, representing 25.52% and 36.94%, respectively. In contrast, a high index of low grade PIN glandular areas (32.81%) and high grade PIN (55.28%) (Fig. 2b, k and l) were observed in the prostate of TC22 (8–12) group. Glandular regions showing well-differentiated adenocarcinoma (2.45%) were verified in the TC22 (8–12) group, (Fig. 2k, arrow). On the other hand, well-differentiated adenocarcinoma represented only 0.95% in the prostate of same aged Nintedanib treated mice. In contrast, the TN22 (12–16) group prostate showed a reduction in high grade PIN (36.68%) and well-differentiated adenocarcinoma incidence (1.94%) in relation to the TC22 (12–16) group (Fig. 2m and n), which presented morphological similarity to the TC22 (8–12) group, considering age similarity. Both TC22 (8–12) and TC22 (12–16) control groups showed hypertrophied prostatic stroma and collagen fiber thickening, due to increased fibrillar elements around the acini (Fig. 2u and x). On the other hand, the same aged Nintedanib treated groups presented a decrease of stromal prostatic hypertrophy (Fig. 2v and z).

### Nintedanib significantly decreased of lesion/tumor proliferation in ventral prostate of TRAMP mice

**Fig. 3:**
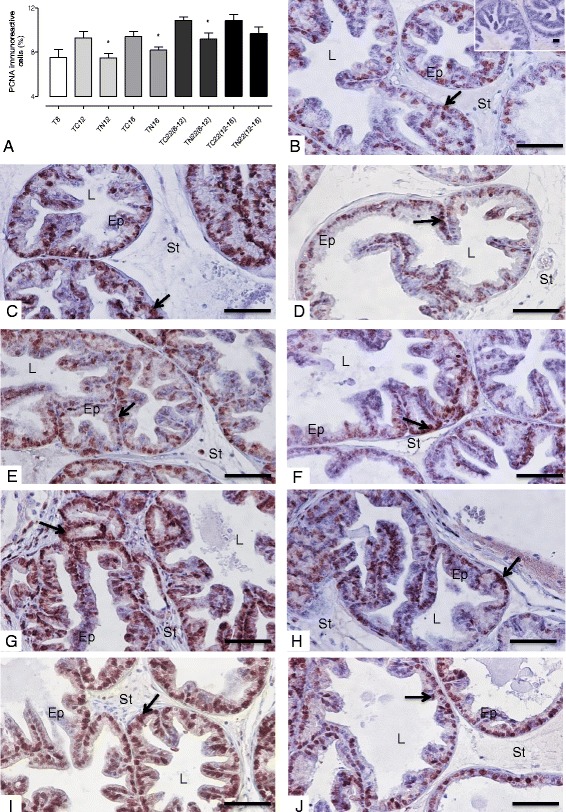
PCNA immunoreactivity. **a** Quantification of PCNA immunoreactivity. **b** T8 group (inset: negative control). **c**: TC12 group. **d** TN12 group. **e** TC16 group. **f** TN16 group. **g** TC22(8–12) group. **h** TN22(8–12) group. **i** TC22(12–16) group. **j** TN22(12–16) group. PCNA-positive staining is indicated by arrow in the nucleus. Values expressed as media or maximum media values and compared between treated and its respective control group. *p < 0.05. Ep = epithelium; St = stroma; L = lumen. Counter-stain: Harris Hematoxylin. 400x, bar = 100 μm

Animals treated with Nintedanib in tumor development grades (8 and 12 weeks of age) and sacrificed immediately, showed a decrease in PCNA immunolabeling in the TN12 and TN16 groups. Furthermore, groups which received Nintedanib from 8 to 12 weeks of age and were sacrificed at 22 weeks of age, also showed a significant reduction of epithelial cell proliferation (Fig. 3), confirming the histopathological analyses.

### Nintedanib intervention inhibited the tumor angiogenesis process in the prostatic tumor tissue in TRAMP mice, despite stopping its treatment

**Fig. 4:**
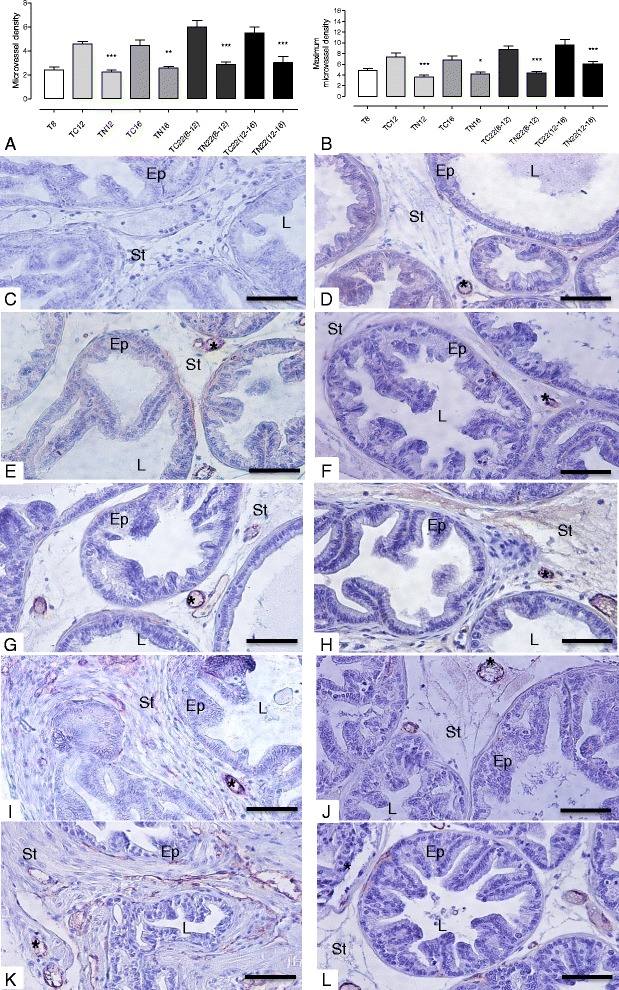
Microvessel density. **a** Mean microvessel density. **b** Maximum microvessel density in a specific field. **c** Negative Control **d** T8 group. **e** TC12 group. **f** TN12 group. **g** TC16 group. **h** TN16 group. **i** TC22(8–12) group. **j**: TN22(8–12) group. **k** TC22(12–16) group. **l** TN22(12–16) Values expressed as media or maximum media values and compared between treated and its respective control group. *p < 0.05; **p < 0.01; ***p < 0.005

**Fig. 5:**
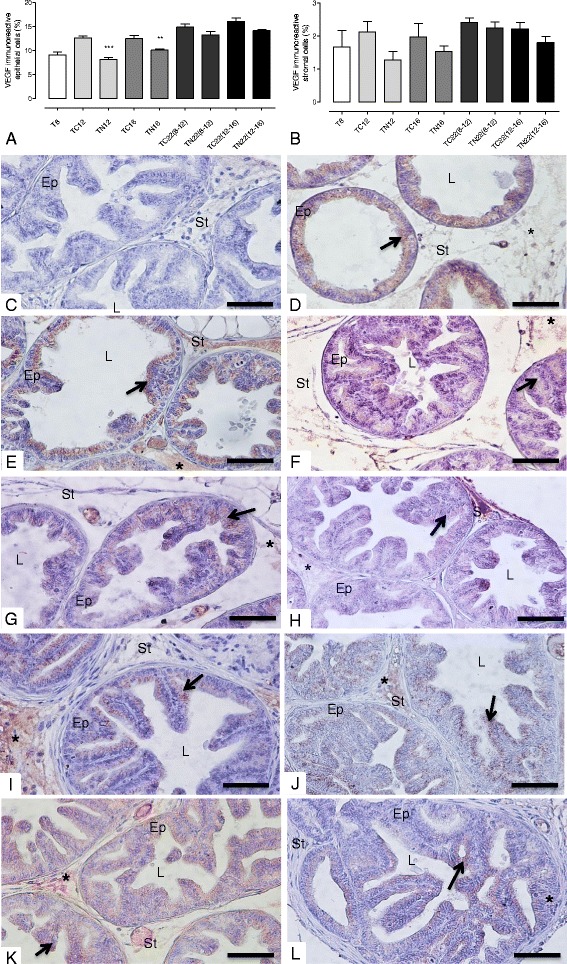
VEGF immunoreactivity. **a** Quantification of epithelial VEGF immunoreactivity. **b** Quantification of stromal VEGF immunoreactivity. **c** Negative control. **d** T8 group. **e** TC12 group. **f** TN12 group. **g** TC16 group. **h** TN16 group. **i** TC22(8–12) group. **j** TN22(8–12) group. **k** TC22(12–16) group. **l** TN22(12–16) group. VEGF-positive staining is indicated by an asterisk in the stroma and arrow in the cytoplasm. Values expressed as media or maximum media values and compared between treated and its respective control group. **p < 0.01; ***p < 0.005. Ep = epithelium; St = stroma; L = lumen. Counter-stain: Harris Hematoxylin. 400x, bar = 100 μm

**Fig. 6:**
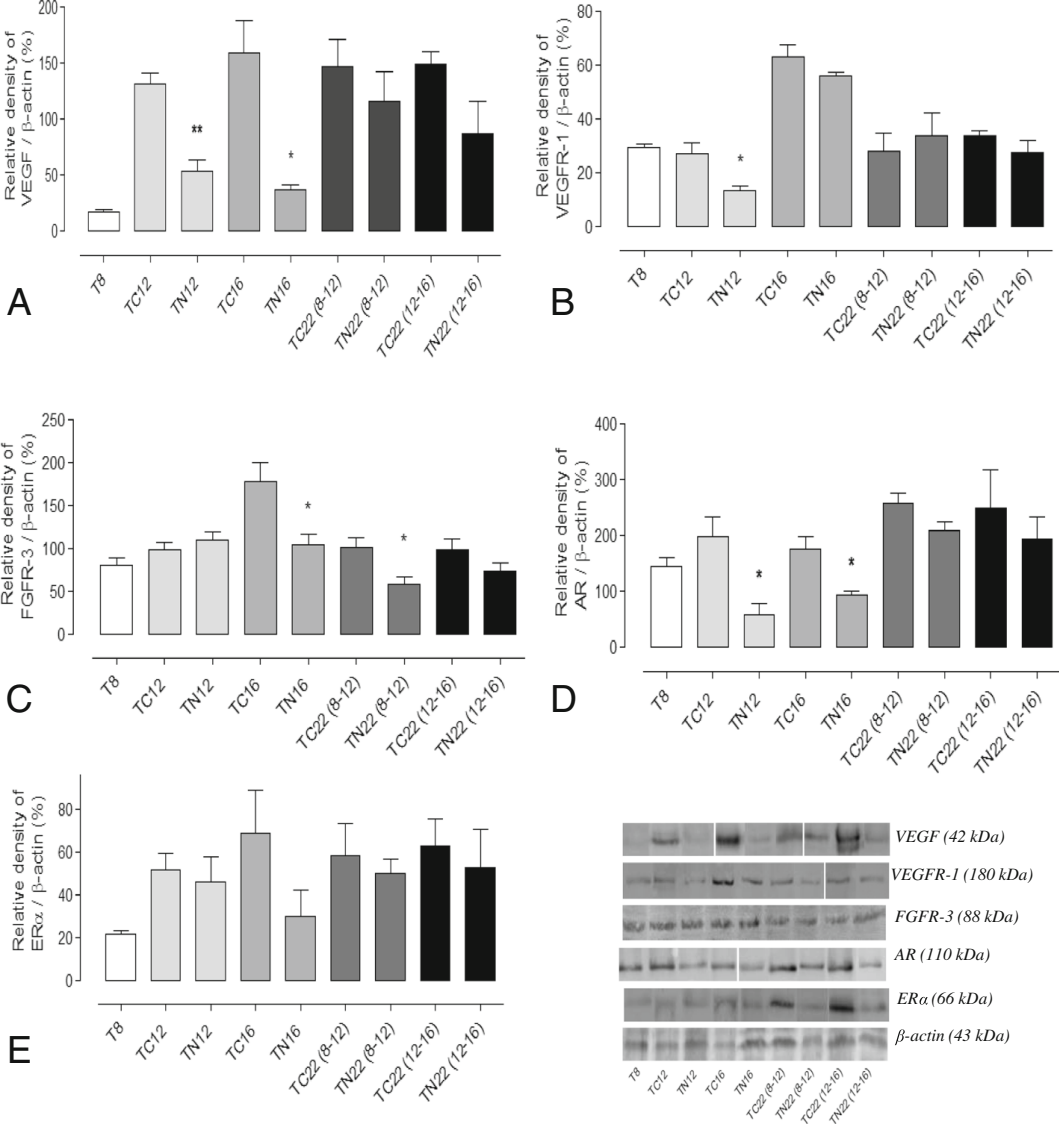
Protein level quantification on the ventral prostate lobe from different experimental groups. **a** VEGF (*n* = 3). **b** VEGFR-1 (*n* = 3). **c** FGFR-3 (*n* = 5). **d** AR (*n* = 3) and **e** ERα (*n* = 3). Values related to β-actin (endogenous control), compared between treated and its respective control group and expressed as media ± S.E.M. *p < 0.05; **p < 0.01

**Fig. 7:**
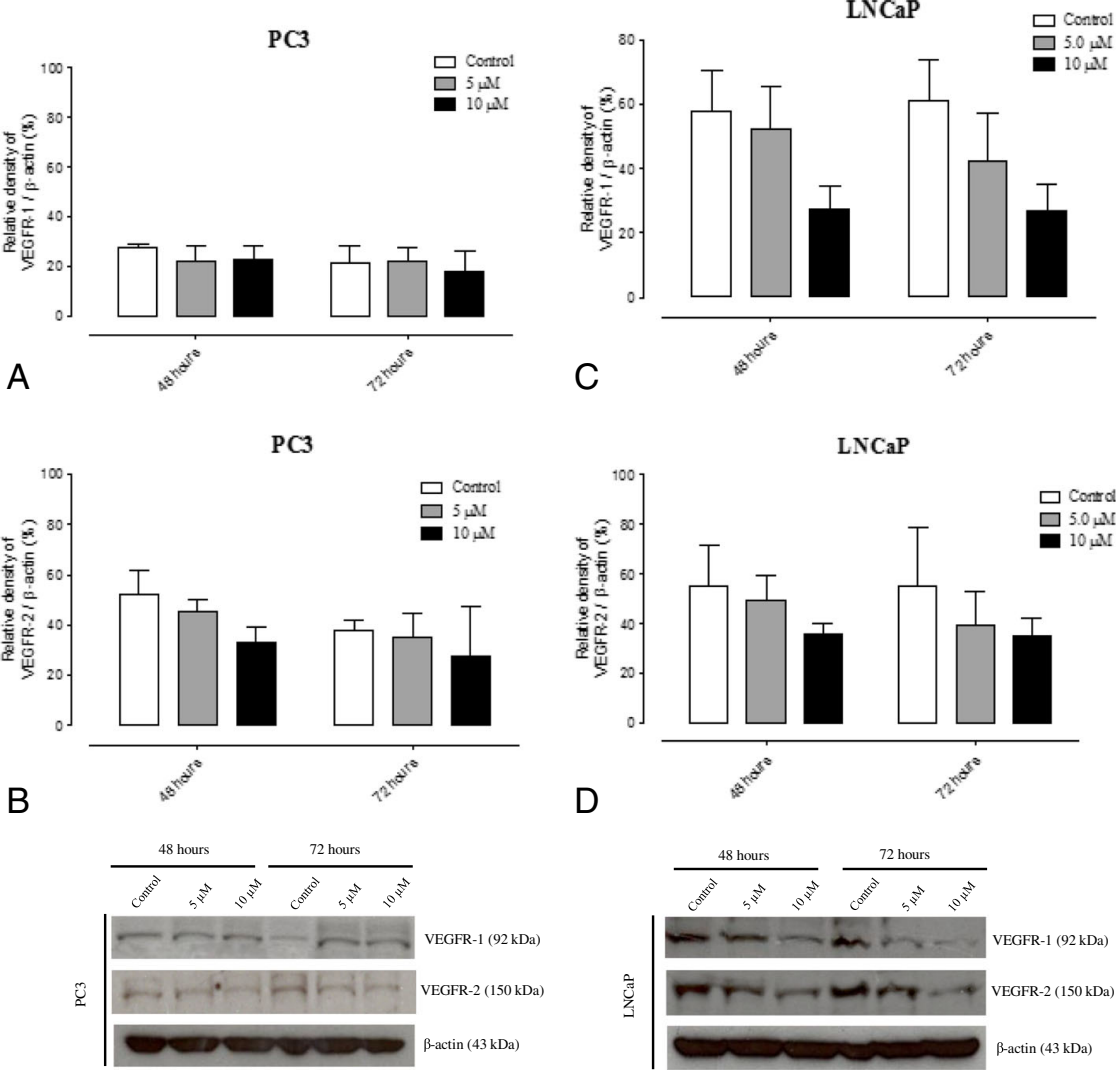
Protein level quantification on PCa cell lines treated with different Nintedanib concentrations. **a** VEGFR-1 quantification in PC3 cells (*n* = 3). **b** VEGFR-2 quantification in PC3 cells (*n* = 3). **c** VEGFR-1 quantification in LNCaP cells (*n* = 3). **d** VEGFR-2 quantification in LNCaP cells (*n* = 3). Values related to β-actin (endogenous control), compared between treated and its respective control group and expressed as media ± S.E.M

Ventral prostates of control TRAMP mice showed a gradual increase in prostatic tumor vascularization, characterized by increase in prostatic microvessel density and epithelial VEGF immunoreactivity. However, in Nintedanib treated TRAMP prostates there was a significant decrease in average and maximum (in a determined spot) microvessel density (Fig. 4). The reduction was stronger in the Nintedanib treated group which started treatment at 8 weeks of age. Also, the Nintedanib treatment groups showed a significant decrease in prostatic epithelial VEGF immunoreactivity in the TN12 and TN16 groups (Figs. 5 and 6a) and VEGFR-1 levels in the TN12 group (Fig. 6b). FGFR-3 maximum expression in ventral prostate was observed at 16 weeks of age in control mice and after which, the expression started to decrease in advanced cancer stages. Nintedanib treatment on the other hand caused a significant decrease in FGFR-3 levels in the TN16 and TN22 (8–12) (Fig. 6c). To confirm whether Nintedanib treatment (5 and 10 μM) could also affect the expression of VEGF receptors in human PCa cell lines, we analyzed the expression of both VEGFR-1 and R-2 in cell lysates. We observed that Nintedanib 10 μM conc. could decrease the expression of VEGFR-2 (although not significant) in PC3 cells at both 48 and 72 h time points, while has no effect on VEGFR-1 (Fig. 7a and b), and also moderately decrease the expression of both VEGFR-1 and R-2 in LNCaP cells (Fig. 7c and d) after 48 and 72 h of treatment with 10 μM was observed.

### Nintedanib treatment led to a decrease in AR expression, but showed no effect on ERα levels

**Fig. 8:**
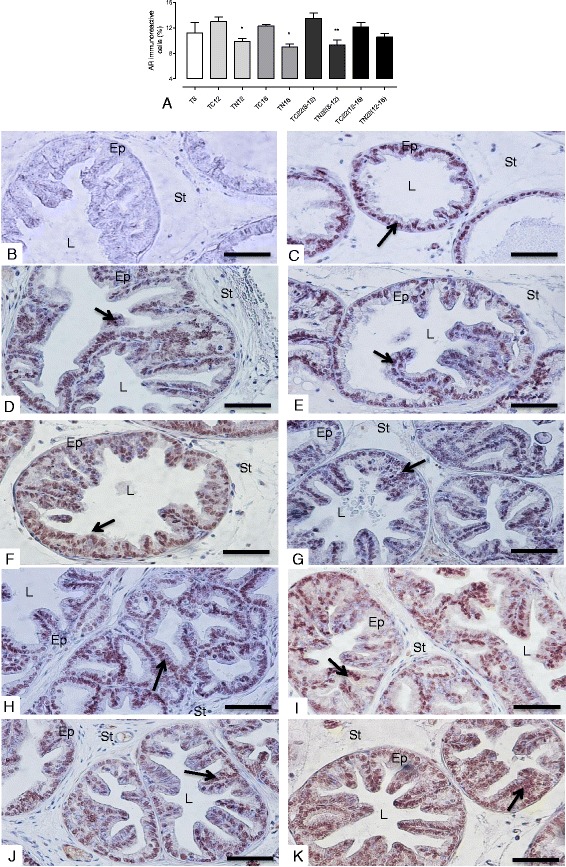
AR immunoreactivity. **a** Quantification of AR immunoreactivity. **b** Negative Control. **c** T8 group. **d** TC12 group. **e** TN12 group. **f** TC16 group. **g** TN16 group. **h** TC22(8–12) group. **i** TN22(8–12) group. **j** TC22(12–16) group. **k** TN22(12–16) group. AR-positive staining is indicated by an arrow. Values expressed as media or maximum media values and compared between treated and its respective control group. *p < 0.05; **p < 0.01. Ep = epithelium; St = stroma; L = lumen. Counter-stain: Harris Hematoxylin. 400x, bar = 100 μm

**Fig. 9:**
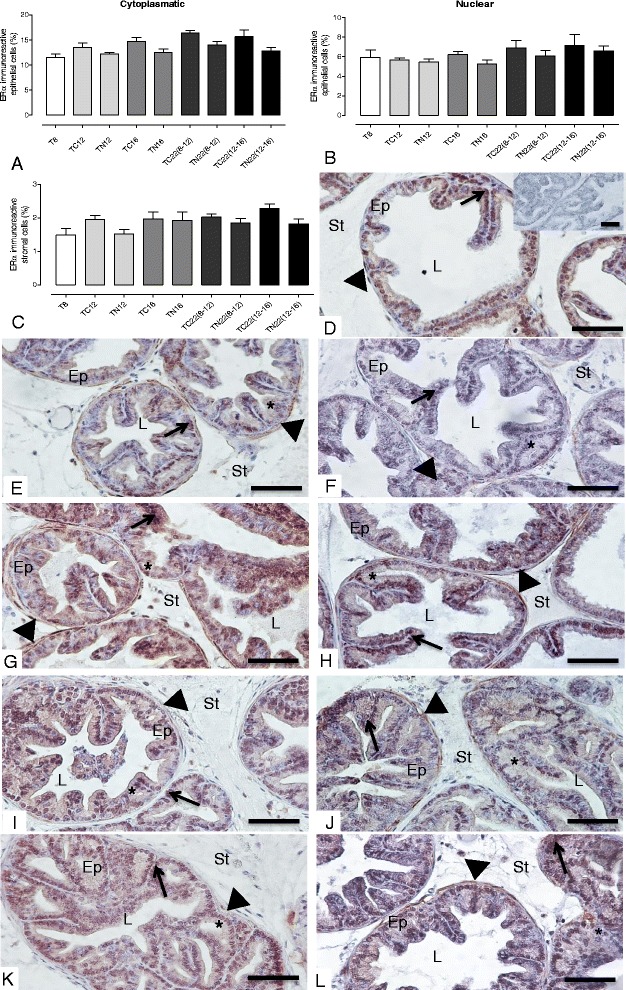
ERα immunoreactivity. **a**, **b** and **c** Quantification of ERα epithelial (cytoplasmic and nuclear) and stromal ERα, respectively. Negative control. **e** T8 group (inset: negative control). **f** TC12 group. **g** TN12 group. **h** TC16 group. **i** TN16 group. **j** TC22(8–12) group. **k** TN22(8–12) group. **l** TC22(12–16) group. **j** TN22(12–16) group. ERα-positive staining is indicated by an asterisk (cytoplasmic), arrow (nuclear) and arrowhead (stromal). Values expressed as media or maximum media values and compared between treated and its respective control group. Ep = epithelium; St = stroma; L = lumen. Counter-stain: Harris Hematoxylin. 400x, bar = 100 μm

The AR immunoreactivity in TRAMP ventral prostate showed a slight increase in the TRAMP mouse prostate from 8 to 22 weeks of age. Notably, Nintedanib treatment reduced not only AR immunoreactivity but also AR protein expression in tissue lysates in the prostate of mice, which began treatment at 8 or 12 weeks of age and were immediately sacrificed (Figs. 6d and 8). While TRAMP prostate also showed a progressive increase in ERα levels in both prostatic epithelial cell cytoplasm and nucleus with increase in mice age, Nintedanib administration had no effect on ERα expression (Figs. 6e and 9).

### Nintedanib treatment neither affected body weight gain nor caused any pathological changes in livers of TRAMP mice

**Fig. 10:**
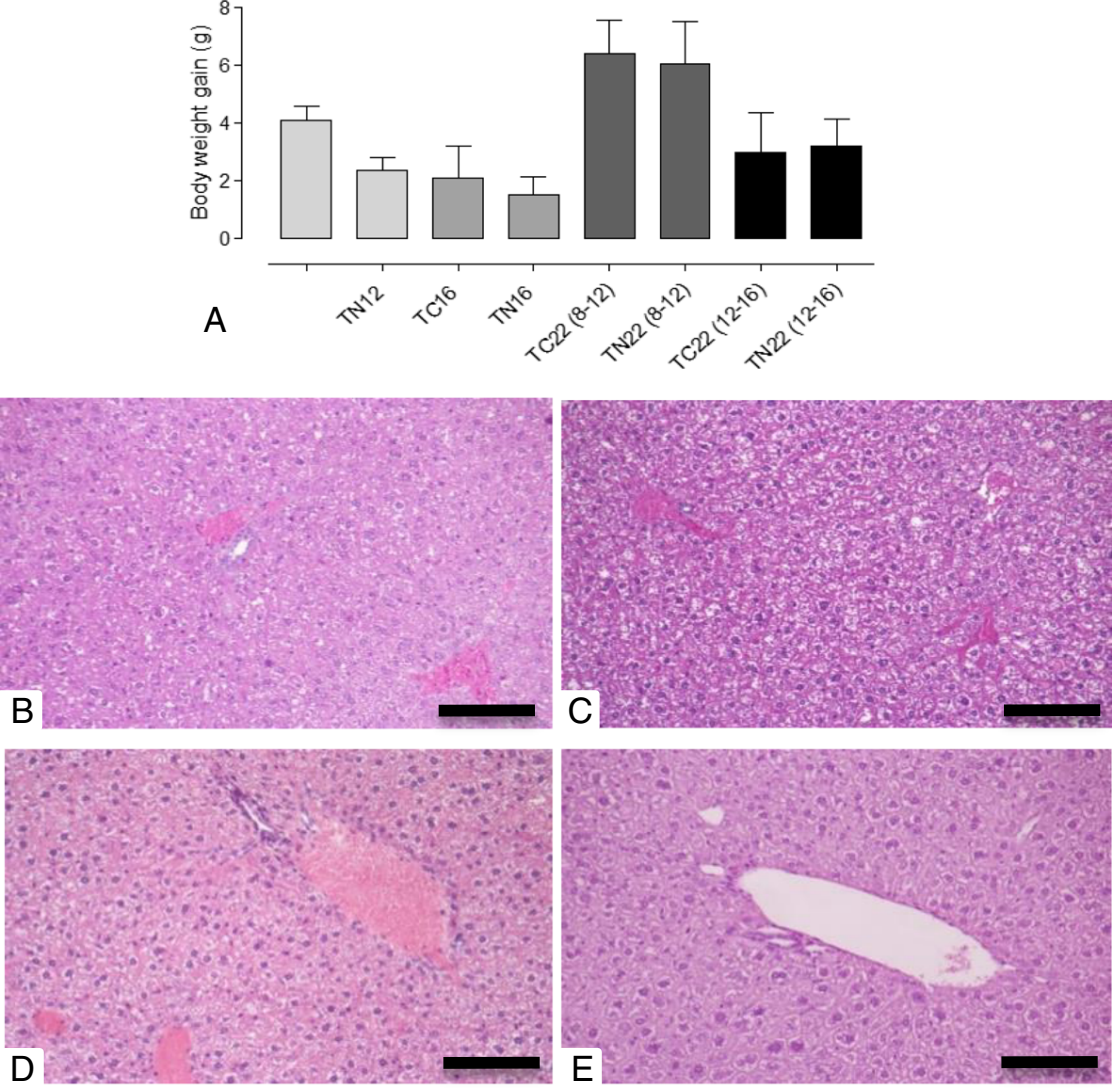
Body weight gain and photomicrography of the liver in TRAMP mice from control and treated groups. **a** Body weight gain of control and treated TRAMP animals. **b** TC12. **c** TN12. **d** TC22(8–12). **e** TN22 (8–12). Normal liver arrangement is noted. The typical hepatic histology with some hepatocytes with large nuclei, indicating polyploidy can be seen in the liver of 12-week-old mice (A and B). Only the presence of polyploid hepatocytes is more frequent in control mice (C) and treated mice (D) in 22 weeks of age. Hematoxylin and Eosin. 200x, bar = 200 μm

The animals from Nintedanib treated groups did not show significant alterations in body weight gain during the treatment when compared to control groups (Fig. 10a). Furthermore, no gross or histopathological changes were observed in the essential metabolic organ liver. Briefly, mice from TC12, TN12, TC22 (8–12) and TN22 (8–12) showed typical healthy hepatic morphology and no changes in ductular structures nor any stromal alterations were noted in the hepatic tissue analysis. Some hepatocytes were bi-nucleated or showed polyploidy (Fig. 10b and c) in the hepatic tissue of the TC12 and TN12 group mice. The bi-nucleated or polyploid hepatocytes were more frequently observed in the hepatic tissues of livers from TC22 (8–12) and TN22 (8–12) groups, but the typical hepatic arrangements were preserved in both groups and no histological differences were detected between them (Fig. 10d and e). No metastatic lesions were found in the livers of the control or Nintedanib treated groups.

## Discussion

It is known that the oncogenic process may cause an imbalance between pro-apoptotic and proliferative signals in the tissues, resulting in uncontrolled cell growth. Cells in a proliferative process demand an increase of nutrients and oxygen, triggering angiogenesis [20]. Angiogenesis is characterized by the formation of new blood vessels from pre-existing capillaries, participating in the critical growth process, progression and tumor metastasis [21]. Also, angiogenesis modulates the expression of several growth factors such as Vascular-Endothelial Growth Factor (VEGF), Platelet-derived growth factor (PDGF) and Fibroblast Growth Factor (FGF), essential for tumor cell growth and survival [22]. These molecules are directly involved in tumor growth and progression, in addition to deregulated cell proliferation, differentiation and survival [23, 24].

Nowadays, studies have shown different prostate cancer treatments with anti-angiogenic drugs involving VEGF inhibition, which is the primary regulator of the proliferation and migration of endothelial cells [25]. Recently, two studies from our research group showed the effects of antiangiogenic therapy, where there was a decrease of microvessel density in TRAMP animals. In addition to this, there was also a reduction of tissue inflammation [26, 27]. However, it is known that there is an occurrence of a compensatory mechanism for signaling exchange between VEGF, PDGF and FGF pathways, as angiogenesis is regulated by multiple factors, resulting in tumor resistance to therapy which only inhibits VEGF [25, 28].

Currently, a new class of triple VEGF, PDGF and FGF signaling inhibitors, known as Nintedanib, is being evaluated. Nintedanib or BIBF1120 has shown good antitumor activity and cell proliferation inhibition in tumor growth due to their associated actions in the tumor cells, endothelial cells and pericytes. An in vitro study showed that Nintedanib treatment induced cell growth interruption and reduced the survival of smooth muscle cells, endothelial cells and pericytes [16]. Furthermore, clinical phase I safety studies showed that 76.2% of patients with different types of solid tumors, following daily treatment with Nintedanib, acquired stability in disease progression and increased average survival time [29]. Moreover, 68.4% of Nintedanib treated patients showed decreased serum levels of prostate specific antigen (PSA) [30]. Recent studies have shown the efficiency of Nintedanib on castration-resistant prostate cancer, the most challenging type of prostate cancer [31, 32].

According to Hilberg et. al [16], Nintedanib effectiveness in delaying tumor progression is accompanied by little or no adverse effects when compared to other anti-angiogenic agents. Thus, based on our results, we concluded that Nintedanib treatment was able to delay prostate cancer progression in mice, especially when this treatment occurred in the initial grades of disease development, at 8 or 12 weeks of age and continued till study end. In addition, the finding herein is important due to the molecular characterization of the prostatic microenvironment, establishing a correlation with clinical Nintedanib trials which are performed in relapsed or refractory cancer [33, 34]. Thus, taking into consideration the results herein, we point to the fact that Nintedanib efficiency could be improved if treatment started in early lesion grades, although these cancer grades are difficult to identify. Importantly, even 10 weeks after stopping the drug treatment, the drug inhibitory effects on prostatic epithelial cell proliferation could be observed.

The animals receiving Nintedanib did not show alterations in body weight gain, reinforcing that there are no toxicity signs after treatment in the study herein, considering that the animal body can provide important information about the animals’ health [35]. Also, the present results showed that liver samples from control and Nintedanib treated groups in both periods analyzed (12 and 22 weeks of age) presented similar characteristics in terms of histology. Thus, there were no treatment-induced changes in liver morphology. The numerous binucleated hepatocytes are a common characteristic in the species, though its meaning is not yet known [36]. However, we found more frequent polyploid cells in the hepatocyte population in 22-week-old animals in both the control and the treated groups. These results suggest that the increase of polyploid hepatocytes was not the result of hepatotoxic processes, as was seen in the study by Gentric [37], where the induction of cellular stress resulted in this feature. On the other hand, it is known that the occurrence of polyploidy increases due to aging in mice [38], which may also vary with the strain [39]. Therefore, this seems to be a likely reason for the polyploid hepatocyte increase in 22 week-old TRAMP mice when compared to those at 12 weeks of age. However, so far there is no characterization of TRAMP mice liver, during the life cycle under healthy or pathological conditions, which allows a comparison with the histological results described in this study. Finally, the results of liver analysis reported here suggest that the concentrations tested in this model are safe. Nevertheless, more extended studies are needed to bring greater clarity to aspects of liver physiology in the TRAMP model, in control conditions or under specific treatment.

Angiogenesis trigger known as "angiogenic switch”, consists of a series of molecular events that can be activated by injuries, inflammatory and immune responses, hypoxia and genetic mutations. In prostate cancer, this trigger seems to occur in the early grades of tumor development in both TRAMP mice and human beings [40].

VEGF is a key molecule for angiogenesis initiation, as its active release signals lead to new vessel formation. Increased VEGF expression is related to tumor formation and progression [41]. A recent study of our research group showed that VEGF as well as the microvessel density increased in 8 to 18-week-old TRAMP mice in the prostatic tissue [26], assessed by CD31 immunoreactivity in endothelial cells. Similar findings were seen in the present study, which showed a progressive increase in VEGF and CD31 in the prostate of 8 to 22 week-old TRAMP mice. The microvessel density analysis by CD31 immunostaining is considered a marker for tumor progression evaluation in several types of cancer [42]. Singh and colleagues [43] showed an increase in microvessel density, shown by the CD31-positive vessel increase, during prostate cancer progression in TRAMP mice. Another study verified that there is also increased vascularization and subsequent tumor growth [44], showing the relationship of angiogenesis in cancer progression, in normal (C57BL/6 J) mice inoculated with TRAMP-C1 prostate cells. Also, according to Pan and colleagues [45] when angiogenesis is inhibited, there is also a decrease in tumor growth. Notably, in all Nintedanib treatment groups there was a decrease in tissue neovascularization, as observed by decreased microvessel density and VEGF immunoreactivity, a crucial molecule for tumor angiogenesis.

Among different pro-angiogenic tumor markers, the most important and well-known are the VEGFs and their receptors (VEGFRs). The VEGFR-1 is present in vascular-endothelial cells and prostatic tumor cells, in addition to hematopoietic cells and monocytes; however, some of the VEGF biological effects on endothelial cells are also given through VEGFR-2, expressed by both prostate tumor cells and stroma [46, 47]. Also, studies have shown that two events are responsible for the triggering of angiogenesis: *the initiation event* in which there is a predominance of the VEGFR-1 expression and the *progression event* showing increased VEGFR-2 expression [48, 49]. Indeed, the results of the current study showed the Nintedanib antiangiogenic activity on reducing the VEGFR-1 levels in TRAMP mouse prostate, the main receptor involved in cell migration and proliferation [50].

The FGFs receptors (FGFRs) also participate in several normal cellular processes of prostatic tissue, such as cell growth and differentiation in addition to angiogenesis. The various receptors found in this tissue differ in their expression, specificity, pathways of activation and effects exerted [51]. Mutations, amplifications and translocations occurring in the genes for FGFRs have also been identified as responsible for abnormal cell proliferation and migration, as well as increasing anti-apoptosis and pro-angiogenic signaling. It is the presence of these alterations that confer tumor sensitivity to the anti-angiogenic factor [52]. Changes in FGFR-3 expression are found in the prostate more frequently in low-grade tumors [53, 54]. This fact can be indicated as an explanation for the peak expression of this molecule found in the present result in 16-week-old TRAMP mice prostate and their subsequent decrease with advancing age when the cancer progresses to a more advanced stage. Indeed, Nintedanib was effective precisely at that time when its expression was increased, since the treatment significantly decreased FGFR-3 levels in 16-week-old TRAMP animals. On the other hand, some studies have shown that FGFR-3 is not overexpressed in benign prostatic hyperplasia as well as in prostate cancer itself [55–57] corroborating our results that showed that the levels of this receptor decrease with the advancement of this malignant disease. Thus, we can attribute the decrease in tumor cell proliferation and subsequent delay in tumor progression to the antiangiogenic effect of Nintedanib, considering the fact there is a decrease in different angiogenic parameters in the prostate of TRAMP mice. This suggests that angiogenesis pathway inhibition may be a clinically useful strategy in the prevention and intervention of PCa progression.

Normal prostate growth and development is androgen dependent, by the binding and activation of testosterone and dihydrotestosterone receptors (AR), leading to cell survival and functional activity [58]. However, the overstimulation of the prostate by these hormones can result in PCa development, showing the central role of ARs in tumor cells growth and survival. Studies in castration resistant cancers, in which there is AR overproduction, result in hypersensitivity to small amounts of circulating androgens, indicating that the overproduction of the receptor contributes to the development and tumor progression [58, 59]. Recently, researchers have shown that a derivative compound of *Magnolia officinalis,* called Honokiol, was able to decrease the viability of androgen-dependent (LNCaP) and independent (C4-2) tumor cells. Also, this could be related to AR expression inhibition, both in these cells and in cells derived from TRAMP mice (TRAMP-C1) [60]. The same authors referred to the PSA regulation by AR, demonstrating that the action of Honokiol in decreasing AR expression was accompanied by the inhibition of their activity. Nevertheless, there was a reduction in PSA secretion by both LNCaP and C4-2 [60]. Another study, using stromal and epithelial cells in AR-knockout TRAMP mice, showed that the lack of expression of this receptor leads to a decrease of tumor size, severity and metastatic invasiveness, and testosterone level reduction. This shows that the AR has a dominant role in promoting proliferation of cancer cells [61]. Thus, the present results are in line with literature reports, which show that AR levels increase slightly in cancer progression in TRAMP mice from 8 to 22 weeks of age. Importantly, we observed that Nintedanib significantly reduced AR levels in the prostate of TRAMP mice, mainly in the group receiving the drug in early tumor development (8–12 and 12–16 weeks of age) and sacrificed immediately, indicating the effectiveness of the drug in inhibiting cell proliferation *via* androgenic pathways.

It is known that the ERα has proliferative effects in PCa and that it participates in more aggressive lesion development, considering that its blockade reduces tumorigenesis [62, 63]. Previous studies have shown that ER levels increased in advanced cancer grades in the ventral prostate of rats and in senile rats when compared to young animals [64], presenting a positive correlation with Gleason score in human beings [60]. In the study herein, we identified a progressive increase in the ERα immunoreactivity with mice age in both epithelial cell nucleus and cytoplasm. On the other hand, Nintedanib treatment showed no direct action on the ERα, suggesting that the Nintedanib protective effect could not occur via the estrogen pathway, at least not directly.

Furthermore, taking into consideration both malignant and non-malignant growth, the uncontrolled epithelial cell proliferation is responsible for glandular volume increase that occurs in the prostate adenocarcinoma of TRAMP mice. Recent studies have shown reduced proliferation of tumor cells to be dependent on the angiogenic pathway after Nintedanib treatment [65–67], corroborating our results that showed reduced cell numbers after treatment of both androgen-dependent (LNCaP) and androgen –independent PC3 human PCa cell lines with Nintedanib.

## Conclusion

Briefly, the results of the study indicate that PCa growth and progression in TRAMP ventral prostate occurs gradually as the age advances. This is partly due to an imbalance of pro- and anti-angiogenic factors in the prostatic microenvironment. Therefore, antiangiogenic therapy could be an effective strategy for targeting PCa. Thus, in our investigative pre-clinical studies in TRAMP model of PCa, Nintedanib, an angiogenesis inhibitor, was able to delay the neoplastic transformation in the prostatic microenvironment, thus reducing tissue vascularization needed for tumorigenesis, which in turn delayed the progression of neoplastic lesions to more advanced stages of the disease. Furthermore, the anti-PCa effects of Nintedanib were confirmed against both androgen-dependent and androgen- independent human PCa cell lines in vitro. Therefore, we can conclude that the Nintedanib antiangiogenic therapy is a promising strategy for both prevention and intervention of PCa, since it is capable of decreasing neovascularization, AR immunoreactivity and delaying tumor progression.
